# Association between average daily gain, faecal dry matter content and concentration of *Lawsonia intracellularis* in faeces

**DOI:** 10.1186/1751-0147-54-58

**Published:** 2012-09-26

**Authors:** Ken Steen Pedersen, Rikke Skrubel, Helle Stege, Øystein Angen, Marie Ståhl, Charlotte Hjulsager, Lars Erik Larsen, Jens Peter Nielsen

**Affiliations:** 1 Department of Large Animal Sciences, HERD – Centre for Herd oriented Education, Research and Development, University of Copenhagen, Groennegaardsvej 2, Frederiksberg C 1870, Denmark; 2 National Veterinary Institute, Technical University of Denmark, Bülowsvej 27, Copenhagen V 1790, Denmark

**Keywords:** *Lawsonia intracellularis*, Porcine circovirus type 2, Quantitative PCR, Pig

## Abstract

**Background:**

The objective of this study was to investigate the association between average daily gain and the number of *Lawsonia intracellularis* bacteria in faeces of growing pigs with different levels of diarrhoea.

**Methods:**

A longitudinal field study (*n* = 150 pigs) was performed in a Danish herd from day 29 to 47 post weaning. Every third day all pigs were weighed, subjected to a clinical examination and faecal samples were obtained. Faecal samples were subjected to dry matter determination and absolute quantification by PCR for *L. intracellularis* and porcine circovirus type 2 (PCV2). Association between average daily gain, faecal dry matter content, numbers of *L. intracellularis* bacteria and PCV2 genome copies in faeces was investigated in a multilevel mixed-effects linear model.

**Results:**

Increasing numbers of *L. intracellularis* log_10_ bacteria/g faeces were significantly associated with decreasing average daily gain (*P* < 0.001). The association was decreasing with increasing faecal dry matter content (*P* < 0.01). The number of PCV2 log_10_ copies/g faeces was not significantly associated with average daily gain of the pigs (*P* > 0.5).

**Conclusion:**

The results suggest a potential application of a PCR quantifying *L. intracellularis* in growing pigs. Faecal dry matter content must be taken into consideration in interpretation of such test results.

## Background

*Lawsonia intracellularis* is a common intestinal infection in pigs and of economic significance in the pig industry worldwide [1]. Pigs may under some circumstances become infected with *L. intracellularis* without reduction in average daily gain (ADG) [2]. Further, pigs can potentially shed detectable numbers of *L. intracellularis* in faeces for eight to 12 weeks [3]. Demonstration of *L. intracellularis* in faeces is therefore not necessarily related to a clinical problem of for example uneven growth in a group of pigs.

Correlation between the number of *L. intracellularis* bacteria in mucosal scrapings and the severity of intestinal lesions has previously been demonstrated [4]. Therefore the number of *L. intracellularis* bacteria in faeces could potentially also be associated to severity of clinical signs, pathology and average daily gain. However, since *L. intracellularis* is an obligate intracellular bacterium and can only be cultivated and maintained in cell cultures [5], little is known about the bacterial number of *L. intracellularis* in faeces [6]. Resent development of quantitative PCR (qPCR) tests for quantification of *L. intracellularis* in faecal samples [6-10] has now made it possible to determine the number of *L. intracellularis* bacteria in faeces on a routine basis. This may be useful for diagnosis of the disease severity in pigs infected with *L. intracellularis*.

The objective of the study was to investigate the association between ADG and the number of *L. intracellularis* bacteria in faeces of growing pigs with different levels of diarrhoea under field conditions.

## Materials and methods

### Design

A longitudinal study (*n* = 150 pigs) was performed in a Danish herd from day 29 to 47 post weaning. Every third day all pigs were weighed, subjected to a clinical examination and faecal samples were obtained. All faecal samples were subjected to dry matter determination. Faecal samples obtained at day one, seven, 13 and 19 were subjected to quantitative PCR (qPCR) testing for *L. intracellularis* and PCV2.

### Selection of herd and batch of pigs

The study was performed in one nursery herd selected at convenience. Criteria for selecting the herd was a history of infection with *L. intracellularis* and PCV2 in nursery and growing pigs, geographic location at Zealand in Denmark and the absence of other major pig pathogens. The selected herd contained 400 sows and 2000 nursery pigs. The herd was classified according to the Danish SPF system [11]to be free from infection with *Actinobacillus pleuropneumoniae*, *Mycoplasma hyopneumoniae*, *Brachyspira hyodysenteriae*, toxin producing *Pasteurella multocida*, porcine reproductive and respiratory syndrome virus, *Sarcoptes scabiei* var. suis, and *Haematopinus suis*. The herd represented a modern intensive Danish herd with a typical modern nursery, applying batch production with all in/all out by room, dry feeding ad libitum based on soybean, barley and wheat, approximately 40 pigs per pen, partially slatted floors and mechanical ventilation with sensors for temperature and humidity with covering in pens. Two weeks prior to the start of the study a serological profile was performed to determine the point of seroconversion for *L. intracellularis*. In each of three age groups (Three, five and seven weeks post weaning) 10 blood samples were collected and examined for *L. intracellularis* specific antibodies by ELISA as previously described [12]. Based on the results of the serological profile a room containing 216 pigs four weeks post weaning were selected for the study. The pigs had not been vaccinated against *L. intracellularis* and had not previously been subjected to antibiotic batch medication. At the first day of the study 10 faecal samples were obtained from pigs with watery faeces to verify an ongoing infection of *L. intracellularis*.

### Selection of individual pigs

On day one of the study all 216 pigs in the selected room were subjected to a clinical examination and a faecal sample was obtained from each pig. Faecal consistency was clinically assessed by one observer using a clinical faecal consistency scale with four categories as previously described [13]. Further, all pigs with a faecal dry matter content below 18% on the first day of the study were considered diarrhoeic [14].Only individual pigs without diarrhoea or any other visual clinical signs were selected for the study. All excluded pigs remained in the pens along with the pigs selected for the study. The pigs selected for the study were ear tagged and their weight was recorded.

### Clinical examinations

During the study period each pig was subjected to a clinical examination, weighed (precision +/− 100 gram) and a faecal sample of minimum 2 gram faeces was obtained by digital manipulation of rectum on days one, four, seven, 10, 13, 16 and 19. On the remaining days pigs were subjected to a visual clinical examination. All examinations were performed by the same observer starting at the same time of day and the pens were examined in the same order. Pigs were re-examined later within the same day if less than 2 gram of faeces was obtained at the examination.

### Treatments

Treatment of pigs was not allowed during the study period. However to apply to the Danish welfare legislation pigs were treated with antibiotics and excluded from the study if they were experiencing depression, dehydration, bloody diarrhoea, necrotic material in faeces and/or poor body condition. Treated pigs were removed form the pens at the day of treatment.

### Sample processing

At the end of each examination day the obtained faecal samples were transported by car to the National Veterinary Institute and stored at 4°C. Processing of faecal samples was performed the following day. Blinding was applied in relation to all laboratory examinations.

### Faecal dry matter

All faecal samples were subjected to faecal dry matter determination. The individual faecal samples were homogenized with a spoon. Faecal dry matter content was determined for minimum one gram of faeces by drying to constant weight using a microwave oven as previously described [14].

### DNA extraction

A suspension of 10% faeces in phosphate buffered saline (PBS) was prepared from each faecal sample. The individual faecal samples were homogenized with a plastic spoon and 0.1 gram of faeces was suspended in 0.9 gram of PBS. The faeces suspension was stored at −80°C until further processing. Total DNA was extracted from the 10% faecal suspensions by using QIAsymphony extraction robot and QIAsymphony Virus/Bacteria Mini Kit (QIAGEN). The protocol was Pathogen Complex 200, the processed sample volume was 200 μl and elution was done in 110 μl. Prior to DNA extraction the 10% faecal suspensions were pre-treated by bead beating in Tissuelyser (QIAGEN) for 20 s and 15 Hz at room temperature with 5 mm stainless steel beads (QIAGEN). The suspensions were centrifuged for 90 s at 6,600 g (MiniSpin plus, Eppendorf) and the supernatant was transferred to the QIAsymphony robot. In each DNA extraction experiment one *L. intracellularis*/PCV2 positive sample and one *L. intracellularis*/PCV2 negative sample were included. The *L. intracellularis*/PCV2 negative sample contained DNA from *E. coli* as determined by qPCR specific for the two included *E. coli* strains (data not shown). The *L. intracellularis*/PCV2 positive extraction control contained DNA from *L. intracellularis* and PCV2 as determined from each specific qPCR (data not shown).

### Quantification by qPCR

All qPCR conditions for *L. intracellularis* were as previously described [10]. For quantification of *L. intracellularis* a standard curve was made from 10% qPCR negative pig faeces and spiked with a 10-fold dilution of the reference strain *L. intracellularis* (ID # 15540) [10]. Each qPCR experiment included the same reference concentrations of pure DNA in triplicate for adjustment of the standard curves to each new qPCR run. The dynamic range for the *L. intracellularis* specific qPCR was 4.3-8.3 log_10_ bacteria/g faeces. The limit of detection was 3.3 log_10_ bacteria/g faeces. All qPCR conditions for PCV2 were as previously described for the PriProET assay [15] except that PCV2 was quantified from a standard curve constructed by spiking a dilution series of PCV2 in 10% faeces. The dynamic range for the PCV2 specific qPCR was 5.0-11.0 log_10_ genome copies/g faeces. The limit of detection was 4.0 log_10_ genome copies/g faeces.

### Statistical analysis

For each of the observation days seven, 13 and 19 it was investigated whether the laboratory results (faecal dry matter content, number of *L. intracellularis* bacteria and PCV2 copies in faeces) were associated to the ADG for the previous seven days in the individual pigs. For each pig three ADG intervals were calculated:

1 ADG day one to seven (kg/day) = (weight on day seven - weight on day one)/6

2 ADG day seven to 13 (kg/day) = (weight on day 13 - weight on day seven)/6

3 ADG day 13 to 19 (kg/day) = (weight on day 19 - weight on day 13)/6

The laboratory results for faecal samples obtained on day seven were tested for association to ADG day one to seven, laboratory results for faecal samples obtained on day 13 were tested for association to ADG day seven to 13 and laboratory results for faecal samples obtained on day 19 were tested for association to ADG day 13 to 19. Therefore each pig provided a maximum of three observations (ADG intervals) to the statistical analysis. The number of *L. intracellularis* bacteria and PCV2 copies were log_10_ transformed before the statistical analysis. Further, the partly ordinal/partly continuous scales for both qPCR tests were taken into consideration. In order to use the continuous part of the qPCR scales a number of new variables were generated. First an ordinal variable for *L. intracellularis* was generated by categorising the qPCR results into zero = *L. intracellularis* not detected, one = *L. intracellularis* qPCR positive below the dynamic range (4.3 log_10_ bacteria/g faeces), two = number of *L. intracellularis* within dynamic range and three = number of *L. intracellularis* above the dynamic range (8.3 log_10_ bacteria/g faeces). A new continuous variable was generated. The values for this variable were equal to the determined number of *L. intracellularis* in log_10_ bacteria/g faeces for faecal samples containing *L. intracellularis* within the dynamic range. For samples below or above the dynamic range this variable was set to zero. Both new variables were included in the same model in the statistical analysis. A similar strategy was used in relation to analysis of PCV2.

Association between ADG, percent faecal dry matter, log_10_*L. intracellularis* bacteria/g faeces and log_10_ PCV2 copies/g faeces were investigated using a multilevel mixed-effects linear model with observation day included as random slopes at pig and pen levels to account for the repeated measures on the individual pigs [16]. The multilevel mixed-effects linear model was favoured over models with different correlation structures by selecting the model with the lowest Akaike’s Information Criteria (AIC), AIC: current model = −286, exchangeable correlation structure = −260, first order autoregressive with random effect for pig = −263, unstructured with homogeneous variances = −283. In the initial model ADG was included as the outcome. Faecal dry matter, the two variables containing the numbers of *L. intracellularis* bacteria and the two variables containing the numbers of PCV2 copies were included as independent variables. Gender and start-weight (weight on day one of the study) were included as co-variables. The initial model was reduced by stepwise backward selection using a *P*-value = 0.05 as the selection criteria. For the reduced model interactions between main effects were investigated. Further, interactions between *L. intracellularis* and PCV2 were investigated. Interactions with a *P*-value below 0.05 were included in the final model. All analyses were performed using the *xtmixed* command in STATA IC version 11.

## Results

At day one 66 of the 216 pigs in the selected room were excluded because of diarrhoea or other clinical signs. Therefore a total of 150 clinical healthy pigs 29 days post weaning were included in the beginning of the study. The pigs were housed in 11 pens, 74 females and 76 males, mean weight = 16.0 kg (standard deviation = 2.55 kg). During the study 10 pigs were excluded because of treatment. Two pigs had to be euthanased. One of those pigs had previously been treated while the other one was euthanased at the end of the study.

**Figure 1:**
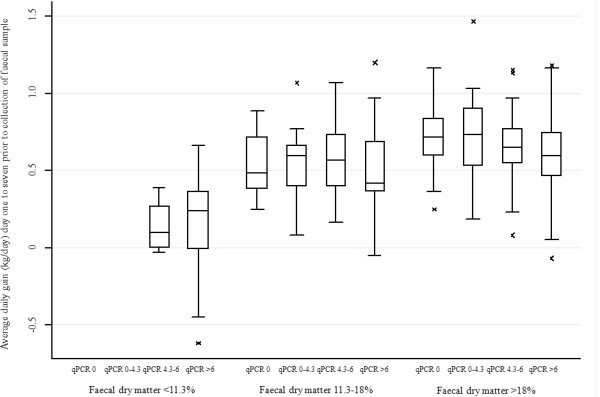
**Association between the number of *Lawsonia intracellularis *bacteria in faeces and average daily gain for pigs with different faecal dry matter content.**

Data was missing randomly on a few other observation days providing a total of 428 observations for the statistical analysis. ADG, faecal dry matter content, numbers of *L. intracellularis* bacteria and PCV2 genome copies in faeces for each observation day are displayed in Table 1. Association between numbers of *L. intracellularis* bacteria and ADG for the previous seven days prior to collection of the faecal samples is displayed in Figure 1.

**Table 1:** **Observed association between daily gain, faecal dry matter, *Lawsonia intracellularis *and porcine circovirus**

**Observation day**				***Lawsonia intracellularis (log***_**10**_** bacteria/g faeces)**		**Porcine circo virus type 2 (log**_**10**_**copies/g faeces)**	
**(ADG interval)**	**DM%**	**No pigs**	**ADG (sd)**^**b**^	**Positive (%)**^**c**^	**Median**^**d**^	**Positive (%)**^**c**^	**Median**^**d**^
7 (day 1–7)	< 11.3	5	0.043 (0.385)	100	7.0	0	-
	11.3-18	36	0.456 (0.179)	89	5.4	31	6.0
	>18	106	0.538 (0.174)	90	5.5	17	6.9
13 (day 7–13)	< 11.3	9	0.144 (0.277)	100	6.3	33	6.1
	11.3-18	21	0.509 (0.183)	90	5.5	52	5.8
	>18	115	0.644 (0.195)	87	5.5	29	6.7
19 (day 13–19)	< 11.3	8	0.260 (0.322)	100	7.1	25	5.6
	11.3-18	27	0.698 (0.305)	89	6.9	33	5.5
	>18	101	0.803 (0.198)	77	5.7	37	6.0

^a^ The three DM% categories corresponds to watery (<11.3%), loose (11.3-18%) and normal (>18%) faecal consistency (Pedersen et al., 2011).

^b^ Average daily gain in kg/day (sd = standard deviation).

^c^ Detected by qPCR.

^d^ Only positive samples.

**Figure 2:**
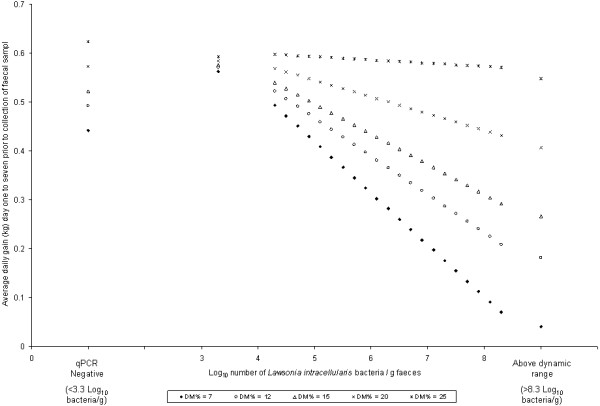
**Predicted values obtained from the multilevel mixed-effects linear model displaying the association between the number of *Lawsonia intracellularis *bacteria in faeces and average daily gain for pigs with different faecal dry matter content (DM%).**

The result of the statistical analysis (multilevel mixed-effects linear model) is displayed in Table 2. An increasing number of *L. intracellularis* log_10_ bacteria/g faeces was significantly associated to decreasing ADG (*P* < 0.001). This association was dependent on the faecal dry matter content as a significant interaction between the number of *L. intracellularis* log_10_ bacteria in faeces and percent faecal dry matter was observed (*P* < 0.01). The negative association between the number of *L. intracellularis* and ADG was increasing with decreasing faecal dry matter content, Figure 2. Further, ADG in pigs negative for *L. intracellularis* was not significantly different (*P* = 0.26) from ADG in pigs with low numbers of *L. intracellularis* (below dynamic range) in faeces. Increasing start-weight was significantly associated to increasing ADG (*P* < 0.001) and ADG was significantly different between the three ADG intervals (*P* < 0.001). Gender (*P* = 0.29) and number of PCV2 log_10_ copies/g faeces was not significantly associated to ADG (*P* > 0.5). Expected ADG (predictive values) obtained from the multilevel mixed-effects linear model for a number of fictive qPCR and faecal dry matter measurements are displayed in Figure 2 for a fixed observation day (day = 19) and start-weight (weight = 16 kg).

**Table 2:** **Modelled association between daily gain, faecal dry matter, *Lawsonia intracellularis *and porcine circovirus**^**a**^

**Variable**		**Coeficient**^**b**^	**Standard**	***P*-value**^**c**^
			**error**	
Outcome	Average daily gain for day 1–7 prior to collection of the examined faecal sample (kg/day)			
Independent	0 = *L. intracellularis* qPCR negative	0^a^		
Categorical	1 = *L. intracellularis* numbers between limit of dection and lower limit of dynamic range	0.18^a^	0.159	
	2 = *L. intracellularis* numbers within dynamic range	0.702^b^	0.279	
	3 = *L. intracellularis* numbers above dynamic range	−0.527^c^	0.177	<0.0001
	0 = PCV2 qPCR negative	0		
	1 = PCV2 copy numbers below dynamic range	0.019	0.048	
	2 = PCV2 copy numbers within dynamic range	−0.009	0.032	0.86
	Gender (barrow/female)	0.025	0.024	0.30
Continuous	*L. intracellularis* bacteria numbers within dynamic range (log_10_ bacteria/g faeces)	−0.144	0.040	<0.0001
	PCV2 copy numbers within dynamic range (log_10_ copies/g faeces)	0.018	0.026	0.50
	Faecal dry matter content (%)	0.010	0.006	0.099
	Observation day (1–19)	0.021	0.002	<0.0001
	Start-weight (kg)	0.034	0.005	<0.0001
Interaction	0 = *L. intracellularis* qPCR negative * faecal dry matter content	0^a^		
	1 = *L. intracellularis* numbers between limit of dection and lower limit of dynamic range * faecal dry matter content	−0.008^ab^	0.007	
	2 = *L. intracellularis* numbers within dynamic range * faecal dry matter content	−0.028^b^	0.013	
	3 = *L. intracellularis* numbers above dynamic range * faecal dry matter content	0.018^c^	0.008	0.0002
	*L. intracellularis* bacteria numbers within dynamic range (log_10_ bacteria/g faeces) * faecal dry matter content	0.006	0.002	0.003
Intercept		−0.36	0.15	
Random^d^	Pen	0.013		
	Pig	0.00004		

^a^For non-significant variables the p-values and coefficients at the time of removal from the model is displayed.

^b^Coefficients with different superscripts within the same variable are significantly different (p < 0.05); ^c^*P*-values for overall effect of variable; ^d^Random effect coefficients displayed as variance.

## Discussion

A SPF herd was selected to avoid any confounding effect of other major pig infections in relation to ADG. Further, a short observation period was selected to minimize the risk of time varying co-variables that could influence ADG during the study.

The ADG in pigs with a low number of *L. intracellularis* bacteria in faeces were unaffected compared to *L. intracellularis* (qPCR) negative pigs. This is in accordance to a previous report that pigs under some circumstances can excrete detectable levels of *L. intracellularis* in faeces without a reduction in ADG [2]. The observed negative association between ADG and numbers of *L. intracellularis* bacteria even in pigs without diarrhoea (faecal dry matter ≥ 18%) supports previous reports of a substantial negative impact of subclinical infections with *L. intracellularis*[17]. However, the association between ADG and numbers of *L. intracellularis* bacteria was dependent on the faecal dry matter content. Similar, decreasing faecal dry matter was associated to decreasing ADG but was dependent on numbers of *L. intracellularis* bacteria. Therefore the effect of *L. intracellularis* or faecal dry matter on ADG can not be assessed in practice without taking both into consideration. With decreasing faecal dry matter the negative association between ADG and *L. intracellularis* was increasing. Interesting in pigs with higher faecal dry matter (<20%) the association between ADG and *L. intracellularis* was minimal. Further, it seems that in pigs with faecal dry matter above 25%, infection with *L. intracellularis* has no negative effect on ADG. This aspect should be further investigated as it has implication for interpretation of *L. intracellularis* PCR test results in practice. A possible biological explanation for this observation could be that pigs with lower faecal dry matter are most affected by the *L. intracellularis* infection and will be more affected by increasing infection severity (as measured by *L. intracellularis* bacteria in faeces).

Vaccination against PCV2 has been reported to prevent PCVAD and thereby reduce the negative impact on ADG [18] and the numbers of PCV2 copies in faeces has previously been correlated to disease severity [19]. PCV2 is widespread in the Danish pig population and it was decided to include examination for PCV2 in the study to explore interactions and handle confounding effects of a PCV2 infection in relation to ADG. The pigs did not have any clinical signs of PCV2 associated disease other than diarrhoea. This may partly explain the lack of association between ADG and the numbers of PCV2 copies in faeces.

In the statistical analysis a retrospective design was applied using the qPCR results and faecal dry matter content at the same observation day to estimate the ADG in the week prior to collection of the examined faecal sample. Other analytic designs were also applied to the same data (data not shown), including testing the association between ADG (measured from day one to 19), total *L. intracellularis* (combining day one, seven, 13 and 19), total PCV2 (combining day one, seven, 13 and 19) and average faecal dry matter (day one, four, seven, 10, 13, 16, 19). These strategies all provided similar results and identical conclusions in relation to the association between ADG, faecal dry matter content, number of *L. intracellularis* bacteria and PCV2 copies in faeces. The retrospective design was selected for the paper, because these results were considered the most useful in veterinary practice. Pig veterinarians are often consulted by farmers for investigation of groups of pigs with diarrhoea and/or reduced performance. The decreasing ADG observed for increasing numbers of *L. intracellularis* bacteria in faeces suggest that determination of the number of *L. intracellularis* bacteria in faeces can be applied for economic assessments of infections with *L. intracellularis*. Further studies are needed to investigate differences between herds and obtain more accurate estimates for the observed associations.

## Conclusions

An increasing number of *L. intracellularis* bacteria in faeces was associated with decreasing ADG in the week prior to collection of the examined faecal sample. The magnitude of the association was increasing with decreasing dry matter content of the examined faecal sample. Therefore faecal dry matter must be taken into consideration for interpretation of the effect of *L. intracellularis*. The number of PCV2 genome copies in faeces was not associated to ADG. No interaction between PCV2 and *L. intracellularis* was observed.

## Competing interest

The authors declare that they have no competing interests.

## Authors’ contributions

All authors conceived and designed the study. KSP and RS performed all clinical herd investigations. ØA, MS, CH and LEL performed all PCR experiments. KSP conducted the statistical analyses. All authors participated in drafting the manuscript. All authors have read and approved the final manuscript.
